# Investigation of Self-Emulsifying Drug-Delivery System Interaction with a Biomimetic Membrane under Conditions Relevant to the Small Intestine

**DOI:** 10.1021/acs.langmuir.1c01689

**Published:** 2021-08-11

**Authors:** Oliver J. Hedge, Fredrik Höök, Paul Joyce, Christel A. S. Bergström

**Affiliations:** † Department of Pharmacy, Uppsala University, 751 23 Uppsala, Sweden; ‡ Division of Nano and Biophysics, Department of Physics, Chalmers Technical University, 412 96 Gothenburg, Sweden; § UniSA Clinical & Health Sciences, University of South Australia, 5090 Adelaide, Australia; ∥ ARC Centre of Excellence in Convergent Bio-Nano Science and Technology, University of South Australia, 5090 Adelaide, Australia; ⊥ The Swedish Drug Delivery Center, Department of Pharmacy, Uppsala University, 751 23 Uppsala, Sweden

## Abstract

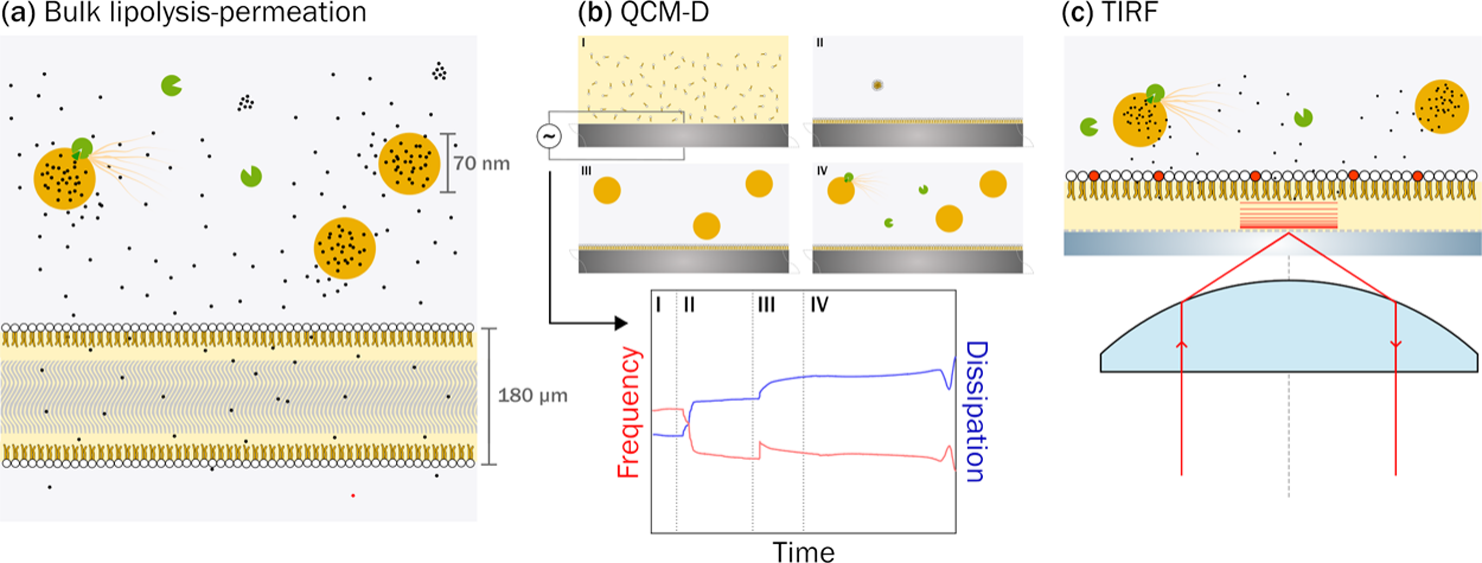

Self-emulsifying drug-delivery systems (SEDDS) have been extensively shown to increase oral absorption of solvation-limited compounds. However, there has been little clinical and commercial use of these formulations, in large part because the demonstrated advantages of SEDDS have been outweighed by our inability to precisely predict drug absorption from SEDDS using current in vitro assays. To overcome this limitation and increase the biological relevancy of in vitro assays, an absorption function can be incorporated using biomimetic membranes. However, the effects that SEDDS have on the integrity of a biomimetic membrane are not known. In this study, a quartz crystal microbalance with dissipation monitoring and total internal reflection fluorescence microscopy were employed as complementary methods to in vitro lipolysis-permeation assays to characterize the interaction of various actively digested SEDDS with a liquescent artificial membrane comprising lecithin in dodecane (LiDo). Observations from surface analysis showed that interactions between the digesting SEDDS and LiDo membrane coincided with inflection points in the digestion profiles. Importantly, no indications of membrane damage could be observed, which was supported by flux profiles of the lipophilic model drug felodipine (FEL) and impermeable marker Lucifer yellow on the basal side of the membrane. There was a correlation between the digestion kinetics of the SEDDS and the flux of FEL, but no clear correlation between solubilization and absorption profiles. Membrane interactions were dependent on the composition of lipids within each SEDDS, with the more digestible lipids leading to more pronounced interactions, but in all cases, the integrity of the membrane was maintained. These insights demonstrate that LiDo membranes are compatible with in vitro lipolysis assays for improving predictions of drug absorption from lipid-based formulations.

## Introduction

Many pharmaceutical compounds now being developed suffer from either poor aqueous solubility, high lipophilicity, or both. For these water-insoluble but cell-permeable compounds, lipid-based formulation can be an effective method for achieving a high absorption while at the same time minimizing absorptive variability between fasted and fed states when administered orally.^1,2^ Self-emulsifying drug-delivery systems (SEDDS) are a class of lipid-based formulations in which mixtures of lipids, non-ionic surfactants, and sometimes co-solvents or co-surfactants form a pre-emulsion concentrate.^3^ When this preconcentrate is dispersed in intestinal fluid, it spontaneously forms a fine emulsion.^4^ The emulsion droplets act as a high-solubility reservoir for the drug, preventing precipitation and conveying the drug to the site of absorption. These actions, together with a rapid equilibrium between the colloidal lipid phase and the continuous aqueous phase from the large interfacial area, prevent the absorption rate from being hindered by the dissolution rate. Thus, SEDDS frequently shows improved oral bioavailability over crystalline, lipid-free formulations for solvation-limited compounds.^5−13^

While facile to assemble, developing SEDDS and translating this knowledge into useful clinical and commercial products has been hindered by a lack of in vitro assays that adequately predict the relative performance of different compositions.^1,14^ The most typically performed test is the in vitro lipolysis assay, in which drug release and digestion kinetics are followed.^15^ However, because of enzymatic lipolysis, the lipids that make up a SEDDS undergo significant transformation after dispersion, as tri- and diglycerides are digested and monoglycerides and fatty acids are absorbed. Alteration of the lipid components affects the colloidal structure of the formulation,^16^ and thus solubility and partitioning of its cargo. A key limitation of the in vitro lipolysis assay is the lack of an absorption step, because absorption of the drug compound is the end-goal of an oral drug-delivery system. For non-colloidal systems, drug release is more easily determined and can often be used to predict the intestinal absorption in vivo; however, drug release from colloidal systems such as SEDDS is notoriously challenging to measure, thus leading to poor predictions when absorption is not accounted for in vitro.^1^

To overcome these limitations, significant efforts have been made to develop new in vitro lipolysis methods that include an absorption step, using cellular monolayers or biomimetic membranes through which the drug can permeate and be assessed after fluxing through this barrier.^17−20^ Keemink et al. demonstrated a significantly improved correlation between in vitro absorption and in vivo plasma concentration for the model drug fenofibrate when absorption was assessed using an in vitro lipolysis-permeation assay with Caco-2 cell monolayers (in contrast to earlier lipolysis assays without an absorption step).^17^ A similar result was demonstrated in the recent study by Klitgaard et al*.,* which showed improved correlations with in vivo absorption for the model drug cinnarizine when assayed in a lipolysis-permeation setup using a biomimetic membrane.^20^

Despite the promise of in vitro lipolysis-permeation assays, it is still unclear whether the biomimetic membrane remains intact under lipase-mediated digestion. In a previous study, interactions between different SEDDS and a biomimetic membrane were suspected during enzymatic lipolysis because flux of the highly permeable model drug fenofibrate increased after 30 min of digestion, which suggests that the membrane did not maintain full integrity.^18^ The artificial membrane employed, lecithin-in-dodecane (LiDo), was an analogue of a well-known commercial product (GIT-0). These products comprise 20% (wt/vol) soy-derived phospholipids in *n*-dodecane, forming a biosimilar membrane when supported by a porous substrate of hydrophobic polyvinylidene difluoride (PVDF).^18,21^ The functional properties of this particular membrane model have, to our knowledge, only been previously described in terms of permeability coefficients,^22^ which means that physical properties and structure when exposed to conditions simulating the dynamic digestion environment of the small intestine remain unknown.

In the present study, we have therefore coupled formulation-dependent drug flux investigations with a quartz crystal microbalance with dissipation (QCM-D) monitoring and specialized fluorescence microscopy techniques to elucidate the effect of SEDDS digestion on membrane structure and integrity. QCM-D can be used to probe the mechanical properties (including mass, density, and viscoelasticity) of a thin film applied to a substrate.^23,24^ Fluorescence microscopy techniques such as total internal reflection fluorescence microscopy (TIRF-M) and fluorescence recovery after bleaching (FRAP) can be used to selectively probe a confined narrow plane of adsorbed membranes (Δ*z* 100–300 nm) to assess membrane integrity and structure.^25−27^ These analytical techniques are widely used to study dynamic interactions on phospholipid bilayers,^28−30^ and were, therefore, selected, in combination with bulk lipolysis-permeation assays, to serve as a novel approach for assessing the effect of SEDDS dispersion and digestion on biomimetic membrane interactions. The insights derived from this study have important implications for the use of newly developed in vitro lipolysis-permeation assays for predicting drug absorption when formulated with lipid-based delivery systems.

## Experimental Section

### Materials

Acetonitrile (≥99.9%), bovine serum albumin (BSA), CARBITOL (diethylene glycol monoethyl ether), dimethyl sulfoxide (DMSO, ≥ 99.9%), d-α-tocopherol polyethylene glycol succinate (TPGS), hexadecane (anhydrous, 95%), Kolliphor RH40 (macrogolglycerol hydroxystearate), methanol (99.9%), olive oil, porcine lipase Type II, Tris-maleate, Tween 85, and warfarin were purchased from Merck (Darmstadt, Germany). Felodipine (FEL) was kindly donated by AstraZeneca (Mölndal, Sweden). MIGLYOL 812 N was kindly donated by IOI Oleo (Wittenberge, Germany). “FaSSIF/FeSSIF/FaSSGF” (simulated intestinal fluid) powders were purchased from Biorelevant.com (Croydon, UK). Lucifer yellow (LY) CH dilithium salt was obtained from Biotium (Fremont, CA, USA). Lecithin 20% Soy PC extract and 1,2-dioleoyl-*sn*-glycero-3-phosphoethanolamine-*N*-(lissamine rhodamine B sulfonyl) were obtained from Avanti Polar Lipids (Alabaster, AL, USA). *N*-dodecane (≥99%) was obtained from Alfa Aesar (Lancashire, UK). Ethanol (99.5%, denatured with 0.4% isopropyl alcohol) was obtained from Solveco (Rosersberg, Sweden). All water used was of grade I from a Milli-Q water purification system (Merck).

### Preparation of the Biomimetic Membrane Solution

The biomimetic membrane solution, LiDo, was prepared by dissolving 2 g Avanti’s Lecithin 20% Soy PC in a 1.5 vol % solution of ethanol in *n*-dodecane. The lecithin was weighed, transferred to a volumetric flask, and solvent was added to make a final solution of 10 mL. The solution was shaken, transferred to a glass vial, and centrifuged (2690*g*, 15 min, 20 °C) to remove the insoluble material. The supernatant was separated into 1 mL aliquots, capped with nitrogen, and stored in a freezer at −18 °C until use. Frozen LiDo aliquots were thawed at room temperature overnight prior to use.

### Preparation and Characterization of Drug-Delivery Systems

FEL was selected as a model drug in this work based on its physicochemical properties. It is well studied, and represents a suitable candidate for formulation in SEDDS on the basis of high lipophilicity, low melting point, limited solubility in water, but high permeability. The innate fluorescence of FEL allows it to be studied label-free in fluorescence microscopy. Formulation excipients and composition (Table 1) were chosen based on their previous use in several publications, both in vivo and similar systems in vitro.^17,18,31^ The excipients are commonly used in the field of lipid-based oral drug delivery. The formulations are representative of different classes of SEDDS but share key similarities that allow for direct comparisons to be made between each formulation. SEDDS were prepared as previously described.^18^ In short, excipients (37 °C) were weighed into 20 mL glass vials in the proportions described in Table 1, capped with nitrogen, vortexed, and shaken overnight at 400 rpm (37 °C). The blank SEDDS were then loaded with FEL (22 mg/g) by mixing and shaking overnight or until complete dissolution.

**Table 1:** Compositions of Drug-Delivery Systems and Properties of Excipients Used^a^

identifier (type)	LFCS category	MIGLYOL 812 N (wt %)	olive oil (wt %)	Tween 85 (wt %)	Kolliphor RH40 (wt %)
F1 (MCT)	I	100			
F2 (s + MCT)	IIIA	40		40	20
F3 (s + LCT)	IIIA		40	40	20
F4 (s)	IV			67	33
*C/D*		8:0, 10:0	16:0, 18:1–2		
*HLB*				11	14–16

a Abbreviations: drug-delivery system types including surfactants (s), medium-chain triglycerides (MCT), or long-chain triglycerides (LCT). LCFS = lipid formulation classification system.^3^ C/D = number of carbons (C) and unsaturations (D) in the acyl chains of the respective digestible lipids comprising the formulation. HLB = hydrophilic–lipophilic balance values according to manufacturer information.

An estimate of the solubility of FEL in the formulations was required in order to select an appropriate dose, where FEL was fully soluble. Previous studies have shown that the solubility of a drug in the formulations can be well estimated from the solubility of the drug in each individual component.^32,33^ Thus, the solubility of FEL in each of the SEDDS components was determined to estimate saturation levels in SEDDS. Solubility of FEL in the F3 SEDDS was experimentally determined to verify this principle in the context of this work.

Equilibrium solubility of FEL in the SEDDS components was determined by adding an excess of FEL (>200 mg/g) to 1.5 mL Eppendorf centrifuge tubes containing solvent (olive oil, Kolliphor RH40, Tween 85, or MIGLYOL 812 N) or SEDDS F3 (control formulation). The mixture was dispersed thoroughly by vortexing, followed by equilibrating under shaking (400 rpm, 37 °C) for 65 h before sampling. The samples were centrifuged (21,000*g*, 15 min, 37 °C) and supernatants were diluted 10× (wt/wt) in a 2:2:1 (vol/vol) solution of acetonitrile, methanol, and sodium acetate buffer (25 mM, pH 5.0), followed by further shaking (400 rpm, 37 °C) for 4 h to fully equilibrate the mixture, an additional 10-fold dilution (vol/vol), and centrifugation. The supernatants of the olive oil and MIGLYOL 812 N samples were transferred to HPLC vials for quantification. Samples from Kolliphor RH40, Tween 85, and the F3 SEDDS were subjected to a third dilution round (10×) and centrifugation step before HPLC analysis due to expected high concentrations. The solubility (*S*) of FEL in each SEDDS was calculated according to eq 1, as previously described.^32,33^
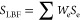
1where *S* is given by the sum of the mass fractions (*W*_e_) of pure solvent multiplied by the equilibrium solubility of the compound in the solvent (*S*_e_).

### Bulk Lipolysis-Permeation Studies

Generally, all experiments were initiated by dispersing 2.8% (wt/vol) SEDDS in fasted state simulated intestinal fluid (FaSSIF), followed by 10 min stirring to generate the emulsified SEDDS. This concentration was selected based on the previous studies involving these formulations.^17,18,31^ The concentration is representative of a realistic scenario in which the loaded formulation is administered to a patient, that is, the amount of formulation required to fill a large capsule and dispersed in a volume corresponding to the fluid volume in the small intestine after intake of a glass of water.^34^ FaSSIF was prepared by dissolving “FaSSIF/FeSSIF/FaSSGF” powder (2.24 g/L) in lipolysis buffer (2/200 mM Tris-maleate, 150 mM NaCl, 1.4 mM CaCl_2_). After 10 min, lipolysis was initiated by addition of porcine lipase extract, reducing SEDDS concentration to 2.5% wt/vol. The extract was prepared by dispersing lipase in cold lipolysis buffer (4 °C, 46 mg/mL), followed by centrifugation (2690*g*, 15 min, 5 °C) and extraction of the supernatant to obtain an extract with an enzymatic activity of 1000 TBU/mL. The final activity of the lipase was 100 TBU/mL in the digestion chamber.

In the bulk lipolysis-permeation experiments—further referred to as in vitro lipolysis-permeation (IVLP)—custom polycarbonate filter holders were used together with commercially available polystyrene 6-well plates (Corning, USA). PVDF filters (pore size 0.4 μm, porosity 0.7, thickness 110–140 μm, Immobilon-P, Merck Millipore) were mounted in the holders and then impregnated with LiDo (16 μL/cm^2^) immediately prior to the experiment. The holders were then transferred into the plate wells and 2 mL of receiver buffer was added to each well. The receiver buffer consisted of phosphate buffered saline (PBS, 10 mM, pH 7.40) supplemented with TPGS (0.2% wt/vol). SEDDS were dispersed in high buffer capacity FaSSIF (200 mM lipolysis buffer, 2 mM sodium taurocholate and 0.75 mM lecithin), supplemented with 10 μM LY. The high buffer capacity was set to prevent the pH from dropping more than 0.2 pH units during lipolysis, while LY permeation was monitored to verify membrane integrity.^18,35^ For all formulations, an initial dispersion was performed by weighing the emulsion preconcentrate into a 15 mL glass vial, followed by dispensing FaSSIF and LY from a 10 mM DMSO stock solution. The mixture was vortexed until no preconcentrate was visible on the glass surface and then transferred to the prepared filter holders (1.5 mL per holder). The plate was placed in an incubating orbital shaker at 37 °C and stirred for 10 min at 450 rpm. The donor media remaining in the glass vial was immediately sampled and filtered through a 0.1 μm nylon syringe-filter to remove precipitated material and larger lipid droplets. Due to the poor dispersibility of F1 formulation, two additional dispersion methods were explored. The first method was directly adding the preconcentrate to the insert containing FaSSIF with a pipet. The second method generated dispersion via high shear using a T18 ULTRA-TURRAX (IKA, Germany) rotor-stator mixer at 8000 rpm for 2 min immediately prior to transfer to the inserts.

After 10 min shaking, lipolysis was initiated by the addition of 165 μL of porcine lipase extract. Samples (200 μL) were taken from the receiver well every 5 min for 20 min, then every 10 min until 60 min of lipolysis had passed. The sampled volume was replaced by fresh receiver buffer following every sampling. At the end of the experiment, samples were taken from the donor media and syringe-filtered. The membrane-supporting filters were subsequently removed from the holders and dried for 3 h at 70 °C. The dried filters were then shredded and equilibrated in 5 mL acetonitrile for 24 h in an orbital shaker at 37 °C with shaking at 400 rpm to extract FEL prior to sampling of the extract. Receiver samples were analyzed using a Spark plate reader (Tecan, Austria) to quantify LY by fluorescence measurement and by HPLC–UV to quantify FEL. Donor and membrane samples were diluted 100× and 10×, respectively, in acetonitrile/water (8:2) and centrifuged (10 min, 21,000*g*) prior to HPLC–UV analysis.

The procedure outlined above was also performed at ambient room temperature (∼22 °C) and without stirring to determine performance under those conditions. In these experiments, the receiver concentrations were expected to be low enough to require measuring by mass spectrometry (MS) instead of UV absorbance, and use of surfactants in MS can reduce signal intensity by ion suppression.^36^ For this reason, the receiver buffer was composed of PBS supplemented with BSA (4 wt %), which has an effect similar to TPGS in that it increases the effective receiver volume by binding to the permeated solute, but it is more easily purged from a solution due to its low solubility in organic solvents. To precipitate the BSA after sample collection, the samples were diluted 3× with cold acetonitrile and centrifuged (21,000*g*, 15 min, 4 °C) prior to analysis of FEL via LC–MS.^37^

### Solubilized FEL and Digestion Kinetics during In Vitro Lipolysis

Due to the restricted volume in the filter holders, supplementary bulk lipolysis experiments—further referred to as in vitro lipolysis (IVL) experiments—were carried out to better capture the dynamics of the aqueous drug concentrations in the donor compartment, as well as capture lipolysis kinetics. In these experiments, 1.14 g of SEDDS was weighed in a jacketed glass vessel and heated to 37 °C in a water bath prior to the addition of 40 mL of low buffer capacity FaSSIF (2 mM). The SEDDS was dispersed by overhead stirring at 400 rpm. After 10 min of dispersion, 4.44 mL of porcine lipase extract was added to commence lipolysis. During lipolysis, pH was kept at 6.5 via autotitration (Pharm Titrando 800, Metrohm, Switzerland) of 0.2 M sodium hydroxide solution. Samples were taken every 3 min of dispersion and every 5 min of lipolysis until 30 min, then every 10 min until 60 min of lipolysis. After sample collection, 5 μL/mL of lipase inhibitor (0.5 M 4-bromophenol boronic acid in methanol) was immediately added to prevent further lipolysis, and the samples were then centrifuged (21,000*g*, 3–5 min, 37 °C) to separate precipitated FEL from the aqueous phase. The supernatants were diluted 100× in acetonitrile prior to analysis with HPLC–UV to quantify aqueous FEL concentrations. At the end of the experiment, NaOH solution was rapidly titrated to increase the pH to nine in order to account for unionized fatty acids and adjust calculation of the extent of digestion.^38^ A blank FaSSIF (no formulation) lipolysis was conducted side-by-side as reference to account for the buffer capacity and digestion of FaSSIF components. The amount of liberated ionized fatty acids (*n*_i_) was then calculated by multiplying the titrated volume by the titrant concentration. Total amounts of liberated fatty acids (*n*_tot_) were calculated by summing total *n*_i_ from pH-stat titration (pH 6.5) with total *n*_i_ from titration of pH 6.5–9 at the end of the experiment, and finally subtracting the corresponding *n*_i_ values from the reference experiments (no SEDDS), according to eq 2.
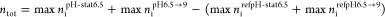
2

*n*_tot_ as a function of time (*t*) during pH-stat titration was then calculated according to eq 3.
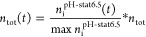
3

Extent of digestion was then calculated as a fraction of *n*_tot_ by the theoretical maximum of fatty acid molecules that could be released during digestion. This maximum was estimated by doubling the molar amount of triglycerides added to the reaction vessel on the assumption that monoglycerides would not be digested within the experimental timeframe due to stereoselectivity of the lipase.^39^

### Quantification of Analytes in Lipolysis-Permeation and Lipolysis Experiments

LY was quantified using a Spark plate reader (Tecan, Austria), set to detection of fluorescence at 428 and 536 nm wavelength for excitation and emission, respectively. FEL was analyzed using a UV-DAD coupled 1290 Infinity HPLC (Agilent Technologies, USA) with a 4.6 × 100 mm ZORBAX Eclipse XDB-C18 column (Agilent Technologies, USA) kept at 40 °C (injection volume 20 μL). The mobile phase consisted of water and acetonitrile (2:8 vol/vol) with isocratic flow (1 mL/min). UV absorbance was monitored at a wavelength of 262 and 360 nm. The retention time was 1.84 min. Sample preparation consisted of 100× dilution in PBS with 0.2% TPGS and a centrifugation step (21,000*g*, 15 min, 25 °C) to purify the matrix.

UPLC-MS analysis was performed using a Xevo TQ MS coupled Acquity UPLC system (Waters, USA) with a BEH C18 column (2.1 × 50 mm, 1.7 μm, Waters). The mobile phase consisted of 5% acetonitrile and 0.1% formic acid in water (solvent A), and 0.1% formic acid in acetonitrile (solvent B). Gradient elution at a constant flow rate of 0.5 mL/min was used. Mobile phase A was decreased linearly (95–0%) from 0.4 to 1.3 min, followed by a constant flow for 0.30 min, and then a linear increase back to 95% A at 1.6 min until the end of the run (2 min, injection volume 10 μL). The column oven and autosampler tray temperature were set at 60 and 10 °C respectively.

The mass spectrometer was operated in positive electrospray mode for FEL and warfarin (internal standard of the analytics). The retention times of these compounds were 1.54 and 1.42 min, respectively. Precursor-product ion pairs followed were *m*/*z* 384 → 278 (cone voltage 5 and collision energy 35 V) for FEL, and *m*/*z* 309 → 163 (cone voltage 22 and collision energy 14 V) for warfarin. Data acquisition and peak integration were performed with MassLynx software (Waters, USA). Sample preparation consisted of dilution 1:2 in ice-cold warfarin in acetonitrile solution (50 nM), and a centrifugation step (4 °C, 20,000*g* for 20 min) to precipitate albumin from the matrix.

Each analysis was performed using minimum eight calibrator samples and four quality controls, in triplicate from different stock solutions. Calibration was performed using first- or second-order weighted linear regression according to best fit. Residual plots were used to detect systematic error from the regression model. A regression coefficient (*R*^2^) > 0.995 was required and the calculated concentrations from the controls were not allowed to deviate more than 10% from the expected concentration (15% for the lowest concentration). Unknown samples with responses outside the calibrated region were not quantified.

### Preparation of PVDF Substrates for QCM-D and Microscopy Experiments

Planar substrates with porous PVDF thin films were prepared based on the protocol developed by Mullen & Euler.^40^ Briefly, PVDF (4% wt/vol) was dissolved in a 9:1 solution of acetone and *N*,*N*-dimethylformamide by sonicating for 3 h at 40 °C. The PVDF solution was then applied to a dried and nitrogen purged silica substrate (QCM-D crystal or glass) by spin coating. A 100 μL PVDF solution aliquot was placed on the sample and allowed to spread by spin coating at 3000 rpm for 60 s. The substrate was dried at 60 °C for 1 min. The nanostructure of the PVDF coating was investigated with scanning electron microscopy (SEM) (JEOL JSM-7800F Prime) by imaging at an accelerating voltage of 1 kV.

### QCM-D Studies

**Figure 1:**
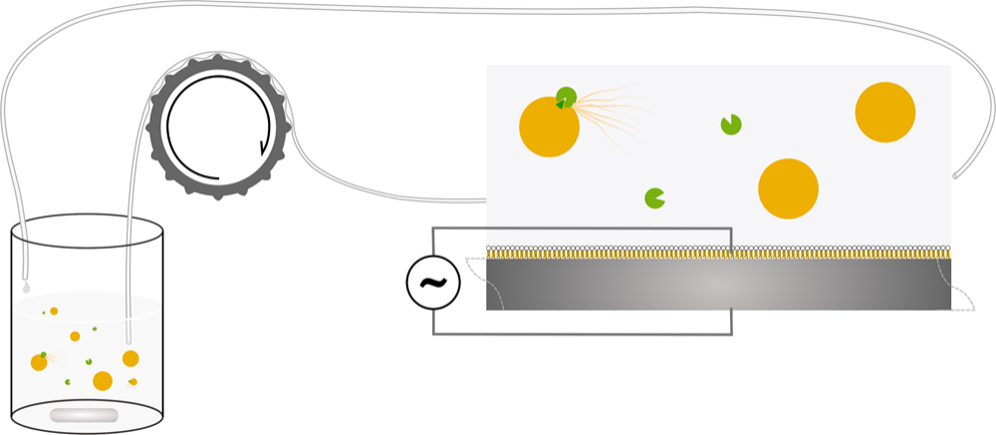
Schematic overview (not to scale) of the QCM-D experimental setup for monitoring SEDDS interactions with adsorbed LiDo membranes during lipolysis. Phospholipid multilayers are here illustrated as the monolayers for visual clarity. A peristaltic pump drives a flow from the reaction vessel (reservoir) to the temperature controlled QCM-D sensor chamber in which interaction with a PVDF-treated sensor chip coated with the LiDo solution was recorded. The outflow from the sensor returns to the reaction vessel, thus creating a closed loop. Orange circles are SEDDS, lipases are in green.

QCM-D measurements were performed on silicon dioxide QSX 303 QCM-D sensors, coated with a PVDF thin film, and mounted in a Q-Sense E4 system (Biolin Scientific AB, Sweden). Sensor chambers were maintained at 37 ± 0.1 °C for the duration of the experiments and the third, fifth, and seventh harmonics were recorded simultaneously to observe changes in frequency (*f*) and dissipation (*D*). The sensors were first flushed with ethanol in *n*-dodecane solution at a flow rate of 50 μL/min for 5 min (or until frequency and dissipation stabilized). Membrane formation was monitored for ∼10–15 min by incubating the sensor chamber with LiDo solution at continuous flow. The adsorbed membrane was then exposed to each emulsified SEDDS in FaSSIF medium for 5 min prior to the addition of porcine lipase to simulate digesting conditions for 60 min, allowing the influence of the emulsion structure and lipid digestion on membrane integrity to be monitored. For this monitoring, a closed-loop lipolysis QCM-D experimental setup was used to enable continuous flow of the digestion medium over the supported lipid membrane (Figure 1). Hence, lipolysis media (5 mL) simulating intestinal digestion conditions (equivalent to those during in vitro lipolysis studies) were cycled through the sensor chambers. The flow rate was set to 50 μL/min for the entirety of the experiment.

### Lateral Membrane Diffusivity Using Fluorescent Recovery after Photobleaching

Fluorescent recovery after photobleaching (FRAP) was conducted on an inverted Eclipse Ti-E microscope (Nikon Corporation) equipped with a perfect focus system (PFS), a CFI Apo TIRF 100× oil objective (NA 1.49), a high-pressure mercury lamp, and an Andor Neo SCC-01322 sCMOS camera (Andor Technology). Lipid membranes were formed on PVDF thin films, supported on a glass microscopy slide (0.13–0.16 mm thickness) and in custom-made ∼100 μL polydimethylsiloxane wells by incubating LiDo solution mixed with l-α-phosphatidylethanolamine-*N*-lissamine rhodamine B sulfonyl (Rh-PE) at a Rh-PE to lecithin ratio of 1:100. A rhodamine filter set (TRITC, Semrock) was used for visualizing the lipid membrane. After ∼10 min, the lipid membrane was rinsed and replaced with lipolysis buffer (50 μL, 200 mM) for a total of five washes to ensure that any unbound LiDo was removed. Validation of the lipid membrane was determined by FRAP in epifluorescence mode, where the Rh-PE lipids were bleached with a Kr–Ar mixed gas ion laser (Stabilite 2018, Spectra-Physics Lasers, Mountain View, CA) at a wavelength of 531 nm. The diffusivity of Rh-PE within the membrane was determined using custom analysis software in MATLAB (MathWorks) by determining the rate of recovery of the bleached hole (as described by Jönsson et al.^41^). The thickness of the supported lipid membrane was determined by quantifying the distance between the glass-PVDF thin film interface and the membrane–buffer interface.

The effect of emulsified SEDDS on membrane integrity was investigated by incubating the PVDF-supported membrane with each formulation at a SEDDS concentration of 3.0% (wt/vol), followed by FRAP analysis. Lipolysis was then initiated by adding a solution of porcine lipase extract (5 μL, 1000 TBU/mL) and FRAP analysis was performed at various time points throughout the 60 min digestion period to determine changes in membrane diffusivity.

### Drug Permeation into the Supported Membrane Using TIRF-M

The effect of emulsified SEDDS under digesting conditions on the permeation of two model drugs across the adsorbed LiDo membrane was investigated by encapsulating FEL and LY within each formulation at a concentration of 0.5 wt %. FEL and LY partitioning, from the aqueous phase to being enclosed in the lipid membrane, was visualized over the 60 min digestion period with a DAPI and CFP filter set (Semrock), respectively. Focus was set at the glass-PVDF interface to ensure that total internal reflection occurred at the deepest point of the lipid membrane. The fluorescence intensity associated with drug permeation was normalized relative to the fluorescence intensity of the drug at the surface in the absence of an adsorbed membrane (i.e., drug that is freely available to adsorb at the surface).

### Data Analysis

Statistical analysis was performed in GraphPad Prism 9 (GraphPad Software, USA) using one-way ANOVA, followed by a Tukey’s multiple comparison analysis test, to compare differences for more than two groups. *P*-values < 0.05 were considered statistically significant. Area under the curve (AUC) was calculated with a Python (version 3.6.5) script by fitting data to Akima splines using scipy.interpolate.Akima1DInterpolator and integrated using scipy.integrate.IntegrateQuad (SciPy version 1.1.0).

## Results and Discussion

### FEL Solubility in the Drug-Delivery Systems

FEL was categorized as freely soluble in the non-ionic surfactants Tween 85 and Kolliphor RH40 and sparingly soluble in the triglyceride-based solvents MIGLYOL 812 N and olive oil, see Table 2. The calculated solubility of FEL in the F3 SEDDS was 21% higher than the experimental solubility (absolute difference: 1.5%), indicating a slight but significant overestimation when using the prediction. Based on these data, the saturation levels of SEDDS loaded with 22 mg/g FEL were 23, 30, and 15% (±0.3%) for F2, F3, and F4, respectively.

**Table 2:** Equilibrium Solubility of FEL in SEDDS Components and Calculated Solubility in the SEDDS^a^

solvent	exp. solubility (mg/g)	exp. solubility (mol_FEL_/mol_solv_)	mole fraction at saturation(mol_FEL_/mol_tot_)	calc. solubility (mg/g)	saturation 22 mg/g FEL (%)
Tween 85	125 ± 1.2	0.598 ± 0.006	0.406 ± 0.006		18
Kolliphor RH40	177 ± 8.7	1.211 ± 0.060	0.594 ± 0.057		12
Olive oil	10.5 ± 0.3	0.023 ± 0.001	0.023 ± 0.001		
F1 (MCT)^b^	27.4 ± 0.5	0.036 ± 0.001	0.036 ± 0.001		80
F2 (s + MCT)^b^				96.4 ± 1.3	23^d^
F3 (s + LCT)^b^	74.3 ± 0.8	0.312 ± 0.003	0.252 ± 0.003	89.6 ± 1.4	30
F4 (s)^b^				142 ± 2.3	15^d^
*Tween 80*	*45.2±4.36*^c^	*0.154±0.015*	*0.139±0.015*		*49*
*Kolliphor EL*	*125±6.23*^c^	*0.782±0.039*	*0.472±0.038*		*18*
*Soybean oil*	*9.59±0.67*^c^	*0.218±0.002*	*0.022±0.002*		
*Captex 355*	*26.4±1.75*^c^	*0.035±0.002*	*0.034±0.002*		*83*

a Solubility for additional SEDDS components (in italics), similar to those included in this study, are included for comparison. Values expressed as mean ± standard deviation (*n* = 3).

b F1 is composed of 100% MIGLYOL 812 N, which is a component of F2. See Table 1 for specifications of the solvents/drug-delivery systems F1–F4, which include surfactants (s), medium-chain triglycerides (MCT), or long-chain triglycerides (LCT).

c From Alskär et al., ref (33).

d From calculated solubility.

The solubility data of FEL corroborate those found by Alskär et al.*,*^33^ in which FEL solubility was determined for similar triglyceride compositions (soybean oil and CAPTEX 355). Relative difference in observed solubility (mg/g) of FEL in MIGLYOL 812 N and olive oil was less than 10% of that in CAPTEX 355 and in soybean oil, respectively (Table 2). The absolute difference was less than 0.1% by molar fraction, which was expected because MIGLYOL 812 N and olive oil contain the same components as CAPTEX 355 and soybean oil, respectively, albeit at slightly different fractions. According to the manufacturer’s information, CAPTEX 355 can contain a higher proportion tricaprylin than MIGLYOL 812 N, but for the most part, the specifications overlap. Olive oil and soybean oil mainly differ in the proportion of monounsaturated to polyunsaturated C18 triglycerides.^42,43^ It could also be predicted that digestion of SEDDS F2–F3 would increase solubility because of the reported higher solubility in mixtures of di- and monoglycerides compared to triglycerides of the same chain length.^33^ Note that this prediction is contingent on the assumption that solubility in the SEDDS components reflects solubility in the emulsion. For the most similar surfactants, the discrepancies were far larger with Tween 85 and Kolliphor RH40, with relative differences in FEL solubility (mg/g) of 177 and 41%, compared to Tween 80 and Kolliphor EL, respectively (Table 2). By molar fraction, the absolute difference was 27 and 12%, respectively. The differing solubility in the surfactants could be interpreted to be a result of lipophilicity differences: the castor oil-derived surfactant studied by Alskär et al. (Kolliphor EL) is more lipophilic than the one studied here (Kolliphor RH40). Furthermore, FEL was shown to be more soluble in mixed di- and monoglyceride compositions than triglyceride compositions.^33^ However, the polyethoxylated sorbitan ester surfactants break this pattern, as FEL had lower solubility in Tween 80 than in Tween 85, but the former could be considered less lipophilic with only one oleate moiety compared to three for the latter.

### Impact of SEDDS Composition on Drug Flux during In Vitro Lipolysis-Permeation Studies

**Figure 2:**
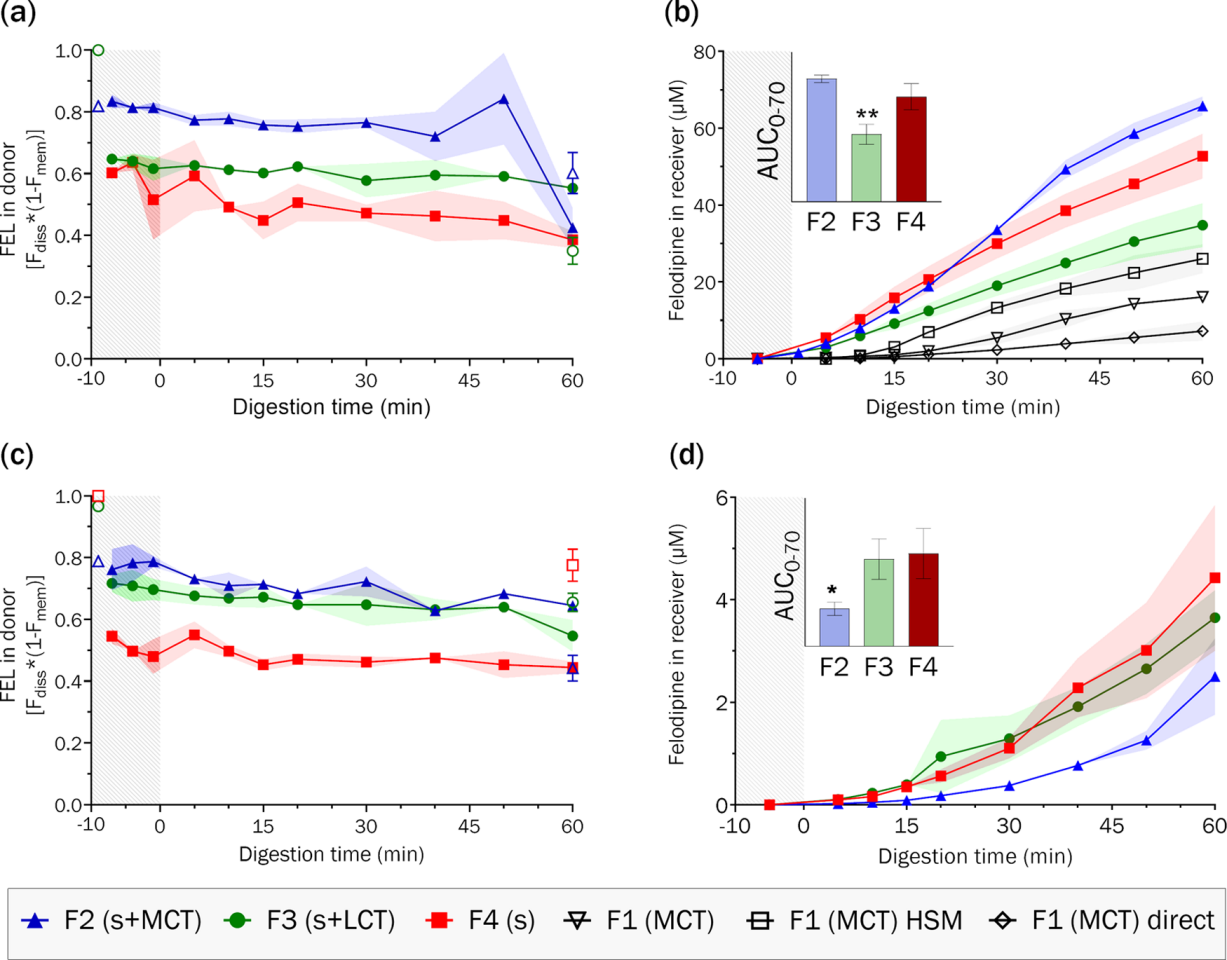
Concentration (μM) of FEL over time profiles of FEL loaded in SEDDS F2 (blue ▲), F3 (green ●), and F4 (red ■) at (a,b) 450 rpm/37 °C, (c,d) no shaking/room temperature. Left panels: Fraction of solubilized FEL (*F*_diss_) during independent in vitro lipolysis experiments (*n* = 2), conducted without absorption but adjusted for membrane absorption (*F*_mem_) determined from separate lipolysis-permeation experiments. Open symbols with colors at −10 and 60 min digestion show *F*_diss_ from lipolysis-permeation experiments (*n* = 3 technical repeats), where membrane binding is built into the model. Right panels: Concentration of FEL (μM) in the receiver compartment (*n* = 3 technical repeats). In (b), SEDDS F1 is shown for different methods of adding the sample: predispersing by vortex mixing (▽), predispersing by high-shear mixing (□), and direct addition to insert (◇). Shaded areas along the curves show standard deviation. Insets show integrated mass-transfer profiles of FEL over 0–70 min, as a measure of relative flux. Asterisk shows the lowest level of significance from ANOVA and multiple comparisons testing of AUCs: *p < 0.05, **p < 0.01.

**Figure 3:**
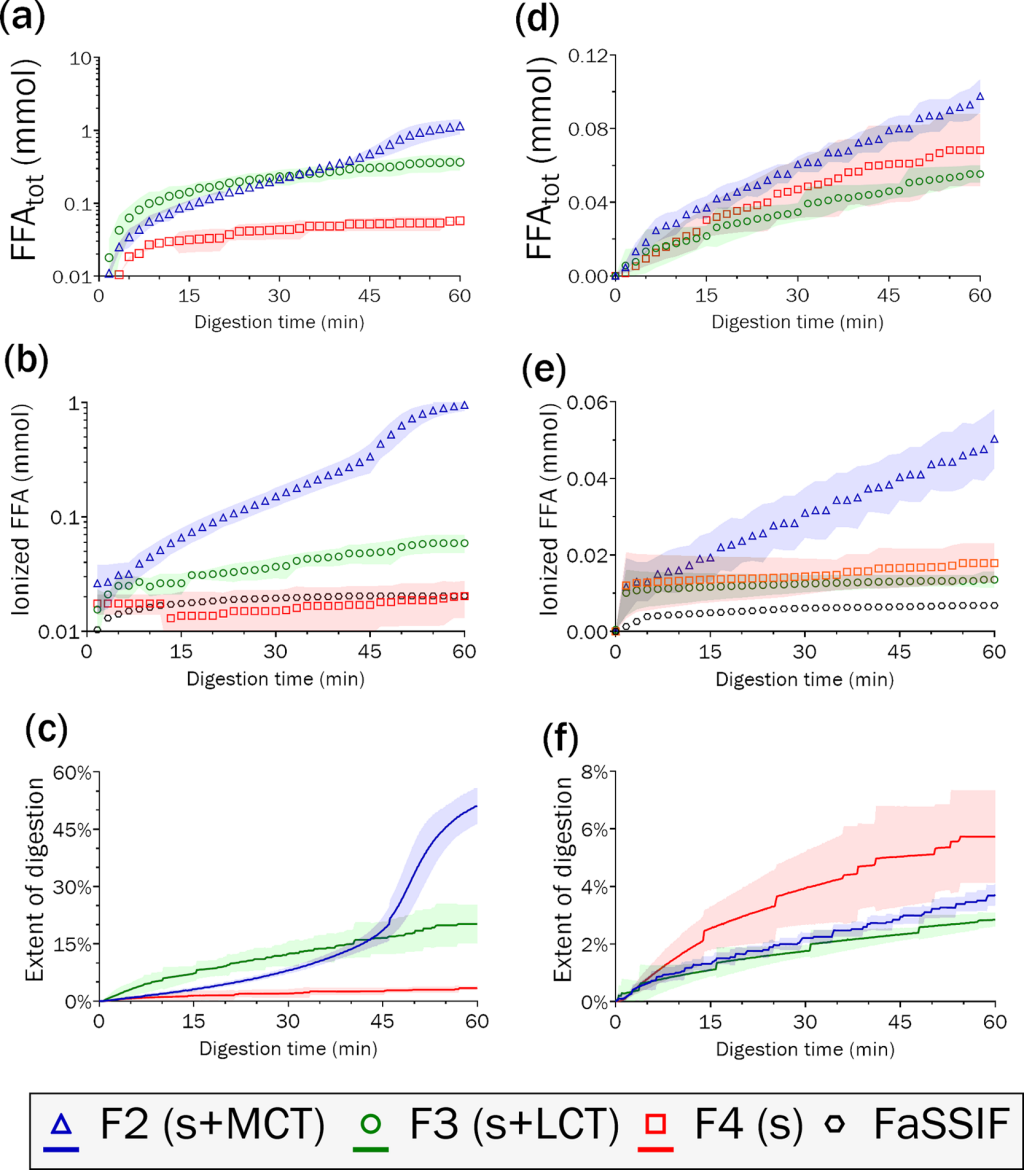
Release of free fatty acids (FFA) from SEDDS F2 (s + MCT), F3 (s + LCT), and F4 (s) when digested by porcine lipase (a–c) at 37 °C, or (d–f) at room temperature in lipolysis experiments without absorption. The total released FFA (top row) was calculated from the ionized FFA directly measured by titration (middle row) by adjusting for the fraction unionized estimated by titration to pH 9 after 60 min to fully ionize FFA, and subtracting the blank digestion medium (FaSSIF). Based on the number of FFA moieties present, an approximate extent of digestion (bottom row) was calculated. Shaded areas represent the standard deviation.

The different SEDDS appeared to have a significant impact on both the fraction dissolved and the mass transfer of FEL across the artificial membrane. During lipolysis at 37 °C without absorption sink, the triglyceride SEDDS (F2–F3) appeared equally capable of keeping FEL solubilized, while the pure surfactant system F4 had a significantly lower solubilizing capacity (Table 3). However, when adjusting the fraction dissolved without absorption sink for the observed membrane fractions from separate lipolysis-permeation experiments, more FEL would appear to be in the aqueous solution in the donor with F2 than with F3–F4 (Figure 2a). The difference for F2 is significant up until the 50 min mark; thereafter, FEL appeared to crash out of solution. This sudden decrease in the fraction dissolved is likely connected to the sharp increase in digestion observed at the same time (Figure 3a–c). As mentioned previously, the solubility could be expected to increase with digestion due to an increased proportion of monoglycerides and fatty acids, and thus the precipitation event is conspicuous. Conceivably, this event is related to a reorganization of the colloidal structure, as the emulsion generated by the F2 SEDDS appeared to crack at the same point, forming two visually distinct phases of different density and opacity (Figure S1a,b).

**Table 3:** Fraction-Dissolved (*F*_diss_) FEL from In Vitro Lipolysis Experiments and Fraction in Membrane (*F*_mem_) from Lipolysis-Permeation Experiments^a^

	*F*_diss_		*F*_mem_		*F*_diss_ × (1 – *F*_mem_)	
SEDDS	37 °C	RT	37 °C	RT	37 °C	RT
F2	93 ± 15	83 ± 7	19 ± 1	14 ± 0.6	75 ± 12	71 ± 6
F3	87 ± 5	81 ± 7	30 ± 0.8	19 ± 1.2	61 ± 3	66 ± 5
F4	52 ± 9	55 ± 5	3 ± 3.6	12 ± 1	51 ± 9	48 ± 4

a Adjusting *F*_diss_ by the apparent amount not found in the membrane [*F*_diss_ × (1 – *F*_mem_)] indicates the effect of having a membrane present. Average ± standard deviation over all time points.

Dynamic light scattering data (DLS) obtained for this formulation show a significant change in the droplet size distribution during the digestion of this emulsion (Figure S1a). The dispersion was initially monomodal, with an increasing hydrodynamic diameter of droplets from 42 nm prior to lipolysis up to 65–76 nm during the first 5% of digestion. The droplet size decreased to 40 nm after 10–15% digestion, possibly stemming from the generation of vesicles or mixed micelles from the digested surface of the droplets. At 20% digestion (∼45 min), the distribution became bimodal as portion of the emulsion droplets appeared to swell to 257 nm. This bimodality coincided with the observed decrease in solubilized FEL and increase in the rate of digestion. Finally, at 30–50% digestion (∼50–55 min), the emulsion seemed to crack, appearing turbid with stirring and phase separating at rest (Figure S1b). At this point, the larger droplets appeared to coalesce and give rise to a trimodal distribution with droplets from 40 nm to several μm. No significant change in appearance or droplet size was observed for F3, with an average size of 52 ± 11 nm (range 37–70 nm, average PDI 12% ± 4.4%).

The emulsions generated by F2 and F4 contributed to higher mass transfers over the membrane than F3 in IVLP experiments (Figure 2b). Flux could not be calculated explicitly due the non-linearity of mass transfer over time, but a measure of flux across the membrane was obtained by integrating these profiles as AUC. From the AUC, it was clear that F3 was significantly less able to promote flux of FEL. In addition to this, several distinct permeation profiles were generated when FEL was loaded in pure triglycerides (F1), depending on the method of dispersion. In the indirect dispersion method employed for all formulations, F1 produced a significantly lower flux of FEL across the membrane. This reduced flux was probably due to the tendency for the emulsions to cream, leading to a much lower surface area for the lipid phase. A lower surface area will also reduce the rate of lipolysis, as pancreatic lipase is activated at the interface of lipid and aqueous phases.^44^ The reduced droplet surface area seems to affect the absorption rate more than the transfer process, an idea that is supported by the even lower flux when F1 was added to the donor medium directly in the insert. When high-shear mixing was used to disperse F1, the flux of FEL significantly increased, but did not reach that of the surfactant-containing F2–F4 SEDDS.

The correlation between solubilized FEL in the donor (IVL experiments) and the flux across the membrane (IVLP experiments) was generally poor, as expected based on previous studies.^17,19,20^ Because the concentrations of FEL in the continuous phase are unknown, it is difficult to draw conclusions from observed concentration profiles in the donor of either assay format. However, the end-of-experiment solubilized fraction in IVLP experiments corresponded better to receiver AUCs from the same experiments, than the donor solubilization profiles from IVL experiments did. Additionally, the IVLP flux appeared to be negatively correlated with the emulsion droplet size observed in IVL experiments. A recent study by Kabedev et al. explored the interaction of the lipophilic drug substance danazol with a phospholipid bilayer.^45^ Their results indicated that mixed-micelles composed of sodium taurocholate and 1,2-dilinoleoyl-*sn*-glycero-3-phosphatidylcholine (DLiPC) can act as shuttles to deliver the payload directly to the membrane interface, or can also fuse with the membrane. While these interactions may be less important than absorption of drug from the continuous phase, they cannot be accounted for in vitro without the presence of a biomimetic membrane. These results reported here, therefore, serve to reinforce the notion that an absorption compartment is useful when studying digestible colloidal systems.

Under benchtop conditions (no shaking, room temperature), the SEDDS appeared to have different effects on the mass transfer of FEL across the membrane than they did under conditions more closely resembling physiological conditions. The observed solubilization capacity in IVL experiments (Table 3) did not strongly differ despite the reduced digestion (Figure 3d–f). However, after adjusting for membrane-bound fraction from the IVLP experiments, the fraction dissolved with F2 and F3 in IVL experiments was more similar at room temperature (Figure 2c) than at 37 °C (Figure 2a) due to the lower amounts of FEL in the membrane at room temperature, and there were lower relative differences between membrane fractions (Table 3). Despite this, F3 and F4 induced significantly higher mass transfer of FEL across the membrane than F2 at room temperature (Figure 2d).

The absence of increased digestion rate and reduced fraction dissolved for F2 at room temperature in IVL experiments could be due to a lack of colloidal rearrangement within the 60 min timeframe, as indicated by DLS, which showed no difference in the droplet size (46 ± 1.4 nm, *n* = 22) from dispersion to end of experiment for the F2 emulsion. However, the IVLP flux of FEL in F2 increased toward the end of the experiment, more than F3–F4. IVL experiments indicate that the rate of digestion decreased within the first 15 min (0–1% digestion), became constant between 15 and 50 min (1–3% digestion), and then increased toward the end for the F2 emulsion, while continuing to decrease for F3–F4. It would thus appear that an increasing rate of digestion is beneficial for the flux of FEL. In the corresponding 37 °C experiment, a similar relationship between the IVLP flux and IVL digestion rate could be seen initially, but a decrease in flux preceded the observed strong increase in the digestion rate between 15 and 50% digestion. Thus, increasing digestion rates appeared beneficial for the flux of FEL in the medium-chain SEDDS (F2) initially, but detrimental at more advanced stages of digestion. Furthermore, colloidal rearrangement appeared to be an important factor influencing the flux of FEL when loaded in the F2 SEDDS. It should be noted that in the small intestine, lipid digestion products (i.e., fatty acids and monoglycerides) are efficiently absorbed and removed from the system.^46^ In vitro, these products remain in the system, with only a minor fraction of fatty acids being bound by calcium, and thus the effects of intestinal digestion on colloidal rearrangement may be inaccurately portrayed. It is unlikely that the observed cracking of the emulsion from F2 SEDDS would occur in the small intestine, as digestion products would be continuously removed, thus maintaining the increased rate of absorption for longer.

A higher activity of lipase (500–1000 TBU/mL) is typically used for in vitro lipolysis assays.^47^ When it comes to permeation assays involving SEDDS, the activity has much more commonly been 0, with the lipase inhibited prior to contact with absorptive membranes or simply not included. Only a few studies have shown simultaneous lipolysis and absorption in vitro. Two of those were conducted with cell monolayers as absorption barriers and digestion by 12.5 mg/mL of Novozym 435,^6,17^ corresponding to an activity of 50 TBU/mL. More recently, cell-free assays simultaneously combining lipolysis and absorption have been published: two lipolysis-permeation assays,^19,20^ and one biphasic lipolysis assay.^48^ Porcine pancreatin was used in these studies, with an activity roughly corresponding to 800–1300 TBU/mL and thus considerable higher than in our work. To put these numbers in context, the lipase activity of the human fasted intestine has been reported to be around 500–600 TBU/mL.^49^ In these cell-free assays, good correlations to in vivo plasma AUCs were reported but accurate ranking of the formulations was not achieved with the IVLP assays.^19^ Furthermore, ranking and correlation was poorer when using data from the initial 30 min of in vitro assay as compared to 6 h. At these high activities, the digestion is essentially completed within the first 15–30 min. Thus, it might be better to slow the lipolysis down in vitro *w*hen the rate of absorption is much lower than in vivo.

With the LiDo artificial membrane, the risk of decreased membrane integrity at higher levels of lipase activity has not been fully explored. In our previous study, we used 660 TBU/mL of porcine pancreatin for digestion of these same formulations. Flux of fenofibrate was observed to increase sharply in some cases between 30–60 min of digestion of the F2 and F3 SEDDS. No increase in the flux of LY was detected, but the increased flux of more permeable substances might have come from reduced membrane integrity. In that study, like the aforementioned other studies, we were not able to show a good agreement between initial 30 min AUCs and in vivo plasma AUCs. We, therefore, chose a lower activity of 100 TBU/mL in the current study, to reduce the risk of membrane integrity loss as well as possibly leading to better agreement between in vitro and in vivo absorption in this type of assay.

### Investigating Colloidal Interactions with Supported Membranes Using QCM-D

**Figure 4:**
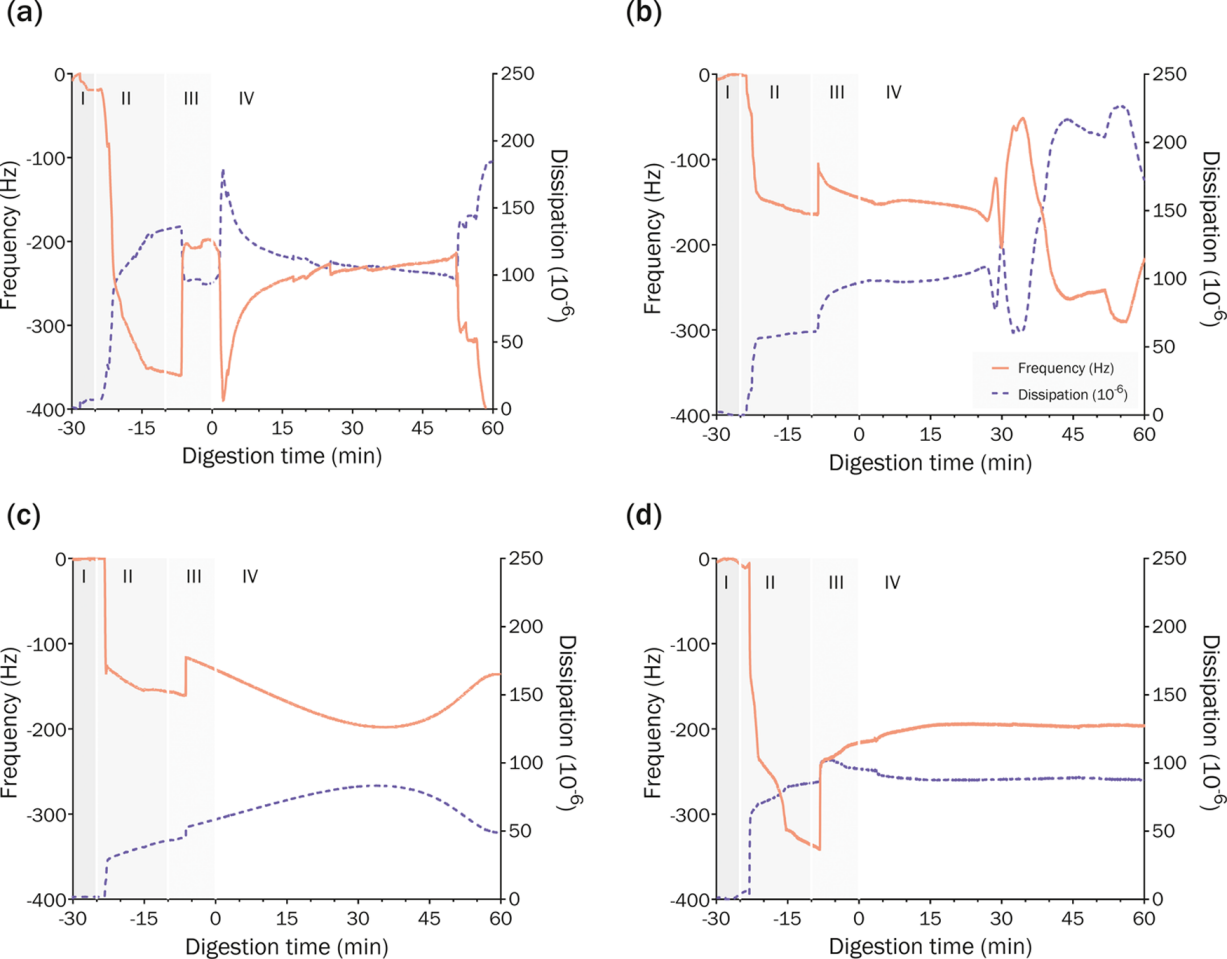
LiDo on the PVDF-treated sensor chip, QCM-D profiles of digestion of drug-delivery systems (a) F1 (MCT), (b) F2 (s + MCT), (c) F3 (s + LCT), and (d) F4 (s). Stages shown in each graph: (I) QCM-D sensor is flushed with *n*-dodecane → (II) multilayer adsorption of LiDo solution onto PVDF surface → (III) emulsified SEDDS in FaSSIF media → (IV) lipolysis was initiated through addition of porcine lipase to the dispersion reservoir. The orange solid lines show changes in frequency (Hz, left *y*-axis) over time, and dashed blue lines show changes in dissipation (10–6, right *y*-axis) over time. Each experiment was performed in independent triplicates, with representative profiles from each condition shown here.

We hypothesized that the colloidal rearrangement of lipid structures during lipolysis was the driving force altering the structure and permeability of the adsorbed membrane and thereby contributing to formulation-dependent changes in drug flux. To investigate this idea, the interactions between each SEDDS with supported membranes adsorbed on nanostructured PVDF thin films (Figure S5) were investigated through changes in frequency (*f*) and dissipation energy (*D*) using QCM-D (Figure 4). Decreased *f* always corresponds to an increase in the mass or density of the adsorbed layer on the sensor, and vice versa, whereas changes in *D* that are not matched by corresponding inverse changes in *f* indicate a change in the viscoelasticity of the adsorbed layer. For example, if *D* increases independently of *f*, this indicates reduced rigidity (increased viscoelasticity) and possible structural changes. However, if such an increase in *D* is matched by a decrease in *f*, this would only indicate increased mass and no change in the viscoelasticity. Alternatively, if *f* increases together with increasing *D*, this would indicate a decrease in mass and reduced rigidity (increased viscoelasticity).

In all experiments, performed in independent triplicates, thick multilayer adsorption of LiDo onto the PVDF surface was identified after 10–20 min by a rapid and significant decrease in *f*, coupled with an increase in *D* (stage II). After 20 min, exposure of the membrane to the emulsified SEDDS in FaSSIF medium (stage III) triggered an increase in *f* and an increase in *D* for all SEDDS, except for F1 (decrease in *D*). This indicates a reduction of mass on the sensor, but also reduced rigidity (increased viscoelasticity) of the adsorbed film. This pattern may be attributed to the surfactant nature of the phospholipids and bile salts within the FaSSIF media that are capable of penetrating and removing amphiphilic membrane components through the formation of micelles and other colloidal structures,^45^ subsequently decreasing the mass and increasing the viscoelasticity of the adsorbed membrane.

Lipolysis was initiated after 10 min of flushing the QCM-D cell with emulsion, to allow for equilibrium with the membrane to be reached. Lipase was added to the reservoir and the experimental setup was shifted to closed-loop QCM-D, allowing continuous flow of the digesting SEDDS medium to the sensor for 60 min (stage IV). Initially, this addition was reflected in only minor changes to *f* and *D* for F2, F3, and F4. In contrast, a sharp decrease in *f* and an increase in *D* were observed for F1 within the first minutes of lipolysis (Figure 4d), which we hypothesize was a result of lipolysis products increasing the overall emulsification of the formulation within the lipolysis vessel. F1 is composed of lipids in the absence of surfactants, and therefore, this formulation emulsifies poorly under non-digesting conditions because the concentration of phospholipids and bile salts in FaSSIF is not sufficient to prevent the separation between the oil and aqueous phases. However, once digestion begins, amphiphilic digestion products (i.e., fatty acids, monoglycerides, and diglycerides) are formed, which aid in emulsifying the lipid components through stabilizing the lipid-in-water interface.^50^ This process was expected to increase the exposure of the lipid component to the adsorbed membrane within the QCM-D cell, thus increasing the interaction (i.e., adsorption) of SEDDS components with the lipid membrane, and indeed, this pattern was observed as evidenced by changes in *f* and *D*. For the remaining formulations, which were well-emulsified due to the presence of surfactants, the immediate change in adsorbed mass and viscoelasticity was not significant upon addition of lipase, because each of these formulations were already well exposed to the membrane.

However, changes in adsorption behavior or membrane structure were evident for F2 after ∼30 min of lipolysis (Figure 4a), through observed dynamic changes in *f* and *D*. The synchronicity of *f* and *D* indicate deposition and removal of mass, but no structural changes in the adsorbed film at this stage. Such changes were not observed for F3 within the 60 min lipolysis period (Figure 4b), but could be seen after 90 min lipolysis (Figure S2). These findings suggest that the adsorption or structural changes observed on the lipid membrane are dependent on lipolysis kinetics, which for F2 and F3 are controlled by changes in the lipid chain length, with shorter-chain lipids (F2) being digested more rapidly. Lipolysis data (Figure 3) support this hypothesis because fatty acid titration kinetics of F2 underwent a significant change in a timeframe similar (*t* = 45 min) to the adsorption or structural changes observed in QCM-D studies. Furthermore, the steady *f* and *D* observed for the nondigestible F4 formulation (Figure 4c), along with the significant changes to *f* and *D* observed after 50 min lipolysis for the lipid-only F1 formulation (Figure 4d), indicate that lipolysis triggers changes to adsorption behavior or membrane structure.

The described events were broadly reproducible in independent replicates (*n* = 3). Coupling these findings with the understanding that colloidal self-assembly of lipolysis products into various liquid crystalline structures is controlled by digestion kinetics,^16^ we can hypothesize that interactions between digestible SEDDS and the adsorbed membrane are mediated through colloidal rearrangements of the lipid components. The exact mechanism of this interaction is not clear, but it is expected that colloidal rearrangement alters the exchange of lipid monomers between the adsorbed membrane and the lipid colloids solubilized within the aqueous media.^51^

To assess whether the dynamic changes in *f* and *D* were dependent on the presence of the supported membrane, QCM-D studies were performed in the absence of LiDo, meaning that digesting SEDDS were exposed to the bare PVDF thin films (Figure S3). Importantly, adsorption patterns revealed similar changes in *f* and *D* after ∼50 and ∼60 min for F2 and F3, respectively. While the timeframes of these *f* and *D* changes do not directly align with changes in the presence of a lipid membrane, these observations support the hypothesis that colloidal rearrangement triggered by lipolysis leads to changes in the adsorption of SEDDS onto surfaces, including both the bare (hydrophobic) PVDF surface and the adsorbed LiDo membrane.

### Impact of SEDDS on the Lateral Diffusivity of Supported Membranes Using FRAP

FRAP analysis was used to quantify the lateral mobility of adsorbed LiDo membranes when subjected to various SEDDS under digesting conditions, to ascertain whether adsorption of SEDDS components (digested and non-digested) affected lipid diffusivity. First, the LiDo membranes were incubated with a small portion of a fluorescent probe (rhodamine B-labeled DOPE), and then the rate of recovery of a photobleached area was monitored (Figure S4). Reduced lipid mobility indicates a ruptured, non-continuous membrane,^41^ and therefore lipid diffusivity is a direct indicator of membrane integrity. FRAP analysis revealed that the adsorbed LiDo membrane remained intact when exposed to digestible SEDDS of all compositions over the course of a 60 min lipolysis period, as evidenced by recovery of the photobleached area.

**Figure 5:**
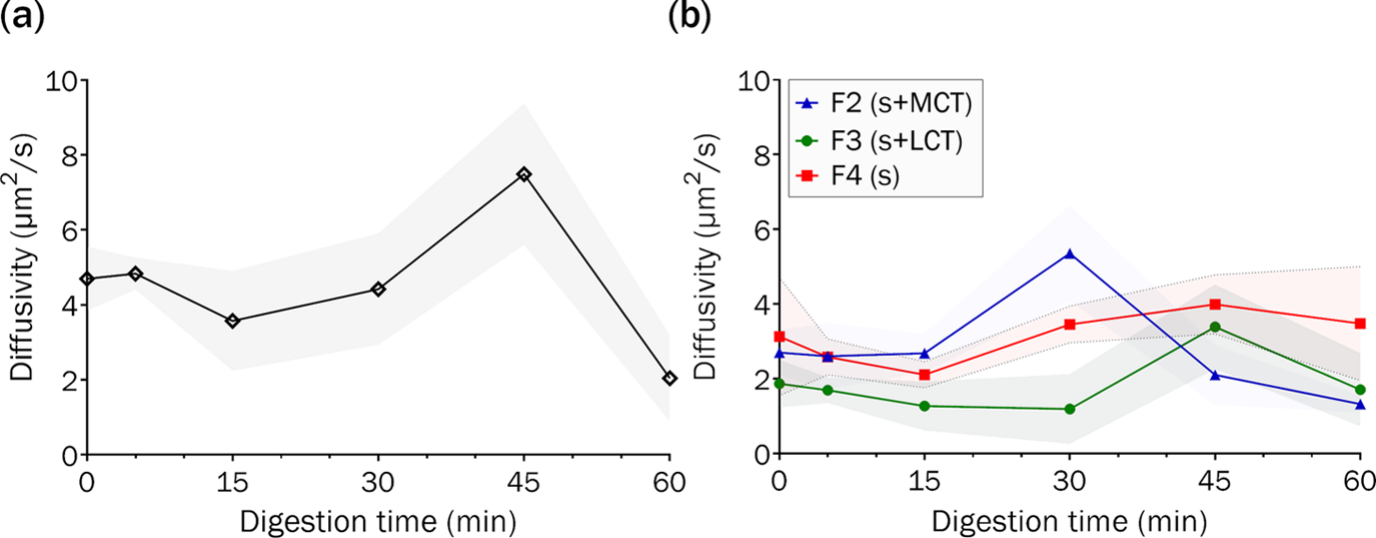
Calculated lateral diffusivity (*xy*-plane) of rhodamine B-labeled DOPE in LiDo membranes, as observed with TIRF microscopy and FRAP. Data are shown as mean and standard deviation (shaded area) of independent replicates (*n* = 3). (a) Diffusivity of the marker with blank digestion medium (FaSSIF) under lipolysis. (b) Different formulations dispersed in digestion medium under lipolysis.

Previous studies have suggested that small changes to the supported membrane structure and composition can trigger alterations to lateral diffusivity.^52,53^ Here, FRAP analysis of the adsorbed membrane exposed to FaSSIF under digesting conditions, in the absence of any SEDDS, revealed fluctuating diffusivities of 2–8 μm^2^/s after 30 min lipolysis (Figure 5a). This finding indicates that membrane exposure to the colloidal systems associated with FaSSIF and hydrolytic lipase enzymes is enough to cause changes in lipid arrangement within the adsorbed membrane. These rearrangements are probably due to the dynamic equilibrium that exists between lipids adsorbed to the PVDF surface and lipids within the bulk aqueous environment, whereby continuous exchange is expected. This dynamic equilibrium is expected to be dependent on lipolysis kinetics and colloidal self-assembly because changes in the composition and structure of lipid colloids alter the expulsion dynamics of individual lipid monomers.^51^

Exposure of the adsorbed membrane to the non-digestible F4 SEDDS revealed negligible fluctuations in lateral diffusivity over the 60 min lipolysis period (Figure 5b), suggesting reduced interaction compared to FaSSIF alone. The presence of surfactants appears to attenuate the effect of FaSSIF components on lateral diffusivity. In contrast, exposing the adsorbed membrane to F2 and F3 under digestion conditions led to spikes in lateral diffusivity at 30 and 45 min, respectively. These findings correlate well with QCM-D observations, where changes in *f* and *D* were shown to be dependent on lipolysis kinetics for F2 and F3. In summary, FRAP analysis in conjunction with QCM-D findings indicates that F4 has little impact on the membrane structure and composition, while F2 and F3 appear to affect the membrane depending on lipid digestion kinetics and subsequent rearrangement of lipid colloids. It should be noted that the FRAP analysis was run at room temperature, which reduced the rate of digestion.

### Impact of SEDDS on Drug Permeation Across the Adsorbed Membrane Using TIRF-M

**Figure 6:**
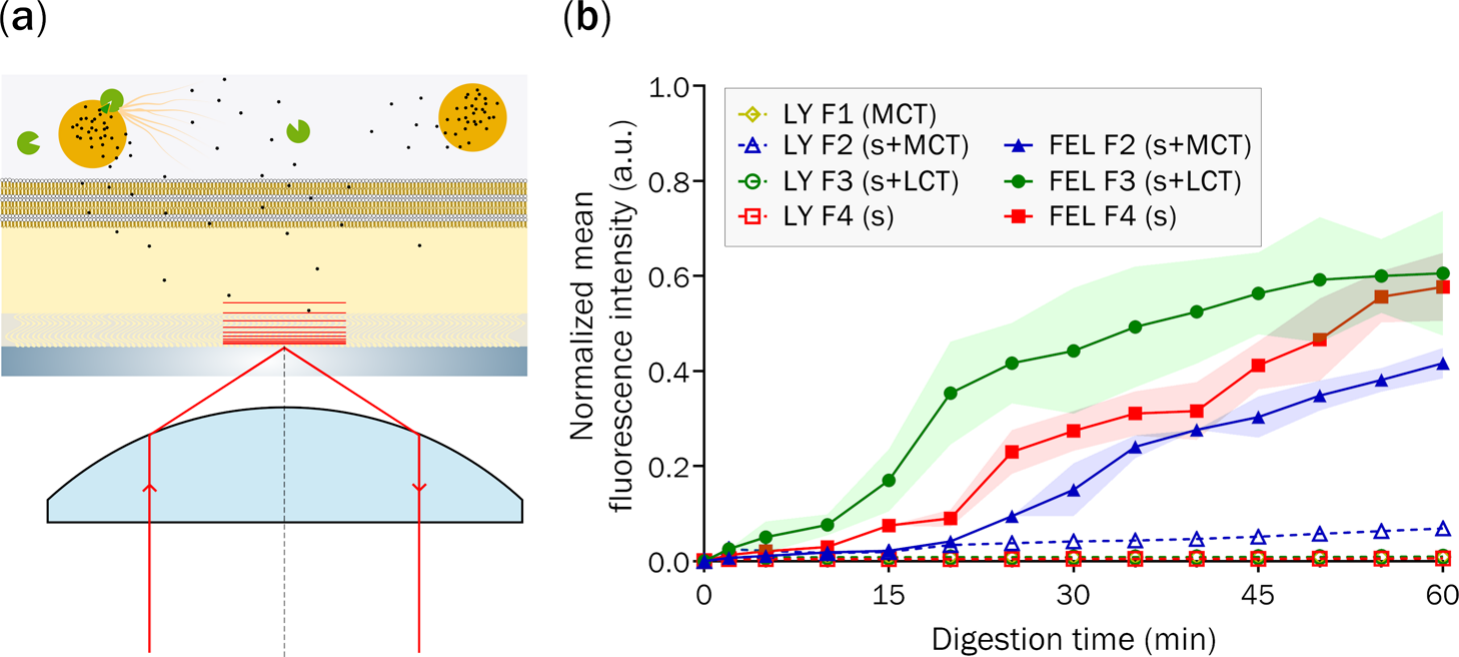
(a) Schematic of the TIRF experiment with the illuminated field represented by the red lines. Legend: SEDDS (orange circles), lipase (green), drug (black dots), membrane (yellow), and filter support (gray). (b) TIRF-M data of normalized mean fluorescence intensity of LY (open symbols) and FEL (closed symbols) at the glass/PVDF interface, basal side of LiDo membranes. FEL was loaded in different SEDDS: F2 (blue ▲), F3 (green ●), and F4 (red ■). Shaded areas show the standard deviation.

TIRF-M presents unique opportunities for monitoring time-dependent drug partitioning and permeation across supported membranes because the evanescent wave created by light reflecting at the interface between a material with a higher index of refraction (i.e., the glass/silica substrate) and a material of lower index of refraction (i.e., PVDF thin film) decays exponentially into the material of lower refractive index.^54^ This means that it is possible to limit the depth of penetration of the evanescent wave to ∼100–200 nm by increasing the critical angle required for total internal reflection. This approach was previously used to monitor FEL permeation across a lipid bilayer supported by a ∼500 nm thick mesoporous silica thin film.^55^ By restricting total internal reflection to within the pores, it was possible to observe the fluorescence associated with only the FEL that diffused across the supported membrane. A similar approach was used in this study, with the LiDo membrane adsorbed onto a porous PVDF support. In the previous study, the pores were ∼7 nm in diameter, allowing the lipid bilayer to be supported above the porous thin film; in this study, the pores of the PVDF support were 0.84 ± 0.1 μm, which meant that the LiDo membrane adsorbed throughout the porous network. Subsequently, it was not possible to monitor drug permeation across the membrane, only drug partitioning within the membrane. However, by confining total internal reflection to the glass-PVDF surface, it was possible to restrict the evanescent wave illumination to a ∼100–200 nm thick subsection within the deepest point of the membrane (Figure 6a). Because the membrane was at least 16 μm thick (calculated by changing the *z*-position between the glass-PVDF thin film interface and the membrane–buffer interface), it can be assumed that any fluorescence detected within the subsection closest to the glass-PVDF interface was available to diffuse across the lipid membrane.

The permeation of poorly permeable LY was first analyzed to serve as a negative control to demonstrate the integrity of the membrane when exposed to each SEDDS under digesting conditions. Importantly, the fluorescence intensity associated with LY diffusion to the glass-PVDF interface was low for all formulations (Figure 6b), indicating that the membrane remained intact when exposed to SEDDS under digesting conditions. F2 had the greatest normalized fluorescence intensity (0.069 ± 0.005 a.u.) of LY diffusion, roughly 7–12 times more intense than the fluorescence of F3 and F4. In sum, it can be concluded that F2 interacted with the membrane to the greatest extent, and thus has a marginally higher potential to affect membrane integrity.

A time-dependent increase in fluorescence intensity was observed for FEL diffusion across the membrane for all SEDDS, with F3 and F4 reaching normalized fluorescent intensities indicating drug diffusion of ∼0.6 a.u. after 60 min lipolysis (Figure 6b). In contrast, the normalized fluorescent intensity of FEL diffusion for F2 was only 0.41 ± 0.03 a.u. This result poorly correlates with the bulk lipolysis-permeation studies performed at 37 °C with stirring. However, strong correlations were obtained between the normalized fluorescence intensity data and bulk lipolysis-permeation studies performed at room temperature without stirring, and so data obtained under static conditions yielded similar results. This result highlights a key limitation of utilizing TIRF-M for analyzing drug partitioning and permeation, and shows that these studies need to be supported with corresponding bulk permeation studies under equivalent experimental conditions. No clear spikes in FEL diffusion were observed, which suggests that the changes in membrane structure as discerned by FRAP analysis (under equivalent experimental conditions) did not lead to changes in drug partitioning and permeation. Most importantly, the complementary nature of QCM-D, FRAP, and TIRF-M strongly indicates that LiDo membranes adsorbed onto nanostructured PVDF substrates maintain their integrity when exposed to the digesting conditions of lipid-based formulations. This result highlights the applicability of these membranes for assessing drug permeation during lipolysis.

## Conclusions

The results from this study reinforce the importance of lipid digestion during in vitro drug absorption assays of SEDDS, as lipolysis will have a significant impact on the observed relative performances. A correlation between FEL flux and lipid digestion kinetics was observed, more so than with a solubilized donor concentration. However, neither drug solubilization nor formulation digestion profiles are likely to be good predictors of intestinal drug absorption from SEDDS; rather, these processes need to be studied in concert. QCM-D and TIRF-M studies revealed differences in formulation-mediated interactions with the membrane, which were amplified by lipid digestion, but importantly, these experiments demonstrated the ability of the adsorbed membrane to remain intact under digesting conditions. The microscopy-based results should be interpreted with caution because the lower temperature restricted the extent of digestion during the experimental timeframe. However, taken together, all results from this study suggest that the LiDo-based artificial membrane is a suitable mimic for the absorption process during lipolysis.

## Supporting Information Available

Additional results from IVLP and IVL experiments, including graphs and photographs; additional results from QCM-D experiments, including graphs with extended timelines for SEDDS F2–F3; graphs of the QCM-D profiles for experiments without the LiDo membrane; micrographs from FRAP experiments; and SEM of PVDF thin film used as the membrane support in microscopy and QCM-D experiments (PDF)
