# Supplementary material for: Investigation of Self-Emulsifying Drug-Delivery System Interaction with a Biomimetic Membrane under Conditions Relevant to the Small Intestine
Source: Langmuir. 2021 Aug 11;37(33):10200–13. doi: 10.1021/acs.langmuir.1c01689 (PMC8388123; doi:10.1021/acs.langmuir.1c01689)
Supplement: Supplementary file 1 — la1c01689_si_001.pdf [file la1c01689_si_001.pdf]

Supporting information

Publication title:

Investigation of self-emulsifying drug delivery system interaction with a biomimetic membrane at conditions relevant to the small intestine

Authors:

Oliver J. Hedge[1], Fredrik Höök[2], Paul Joyce[2,3,4*], Christel A. S. Bergström[1,5*]

[1]Department of Pharmacy, Uppsala University, 751 23 Uppsala, Sweden.

[2]Division of Biological Physics, Department of Physics, Chalmers Technical University, 412 96 Gothenburg, Sweden.

[3]UniSA Clinical & Health Sciences, University of South Australia, Adelaide 5090, Australia.

[4]ARC Centre of Excellence in Convergent Bio-Nano Science and Technology, University of South Australia, Adelaide 5090, Australia.

[5]The Swedish Drug Delivery Center, Department of Pharmacy, Uppsala University, 751 23 Uppsala, Sweden.

*Corresponding authors:
Christel Bergström
Department of Pharmacy
Husargatan 3, Box 580, 751 23 Uppsala, Sweden.
Tel: +46 18-471 41 18 | Fax: +46 18-471 42 23
E-mail: christel.bergstrom@farmaci.uu.se

Paul Joyce
UniSA Clinical & Health Sciences
University of South Australia, Adelaide 5090, South Australia, Australia
Tel: +61 491712756
E-mail: Paul.Joyce@unisa.edu.au

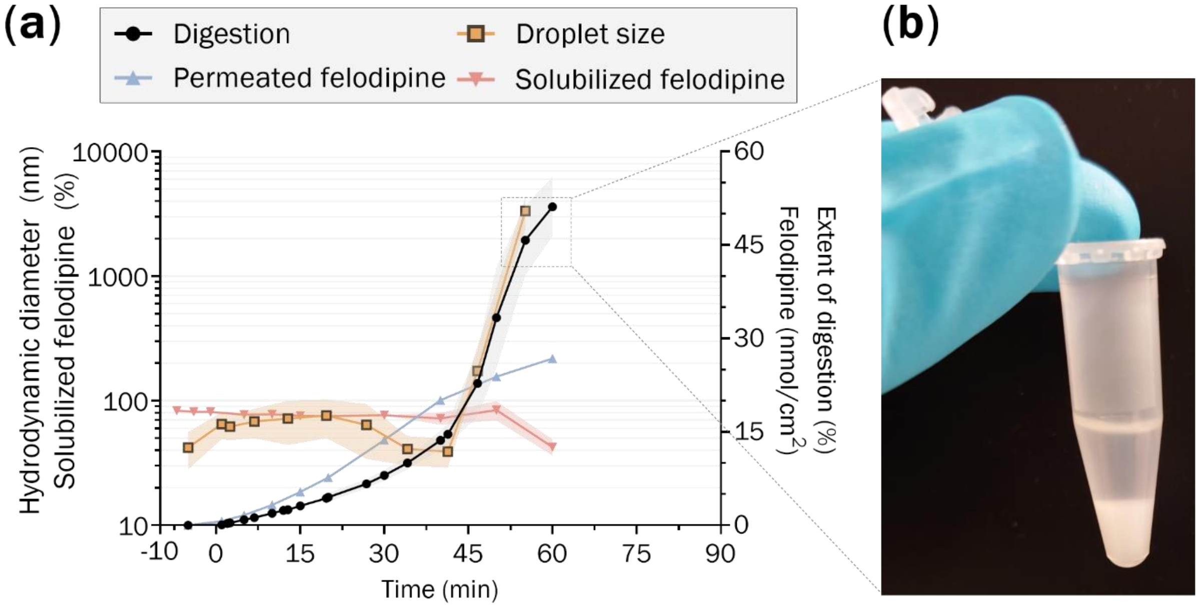

Figure S1. (**a**) Graph showing for SEDDS F2 (s+MCT) at 37°C: the extent of digestion (black circles, right y-axis), hydrodynamic diameter of emulsion droplets (orange squares, left y-axis), permeated felodipine (blue triangles, right y-axis), and solubilized felodipine (red triangles, left y-axis). Particle size distribution was obtained using a Litesizer 500 (Anton Paar, Austria) with a quartz low-volume cuvette (Hellma Analytics, Germany). (**b**) Cracked emulsion from digestion of SEDDS F2 to ~50%.

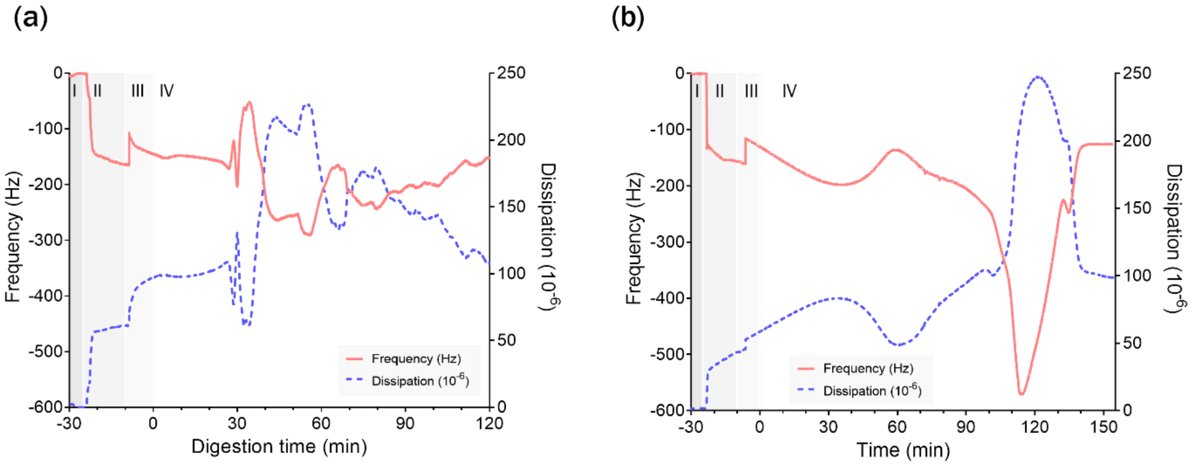

Figure S2. QCM-D profiles of digestion of drug delivery systems (**a**) F2 (s+MCT) and (**b**) F3 (s+LCT). Stages shown in each graph: (I) *n*-dodecane → (II) membrane forming solution (LiDo) → (III) FaSSIF medium with dispersed formulation → (IV) addition of porcine lipase to dispersion reservoir. The red solid lines show changes in frequency (Hz, left y-axis) over time, and dashed blue lines show changes in dissipation (10^{-6}, right y-axis) over time.

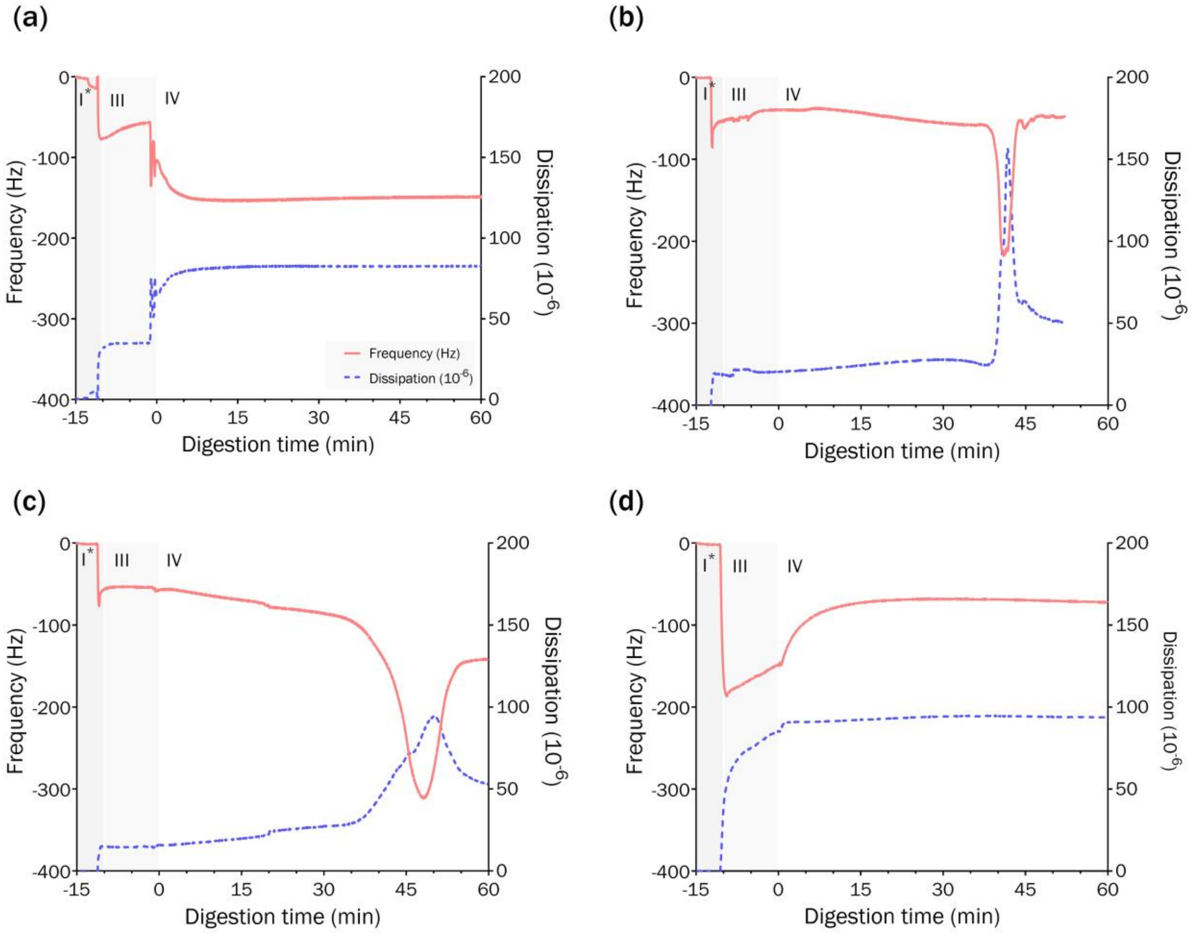

Figure S3. QCM-D profiles showing digestion of drug delivery systems (**a**) F1 (MCT), (**b**) F2 (s+MCT), (**c**) F3 (s+LCT), and (**d**) F4 (s) on bare PVDF-treated sensor chips. Stages shown in each graph: (I*) FaSSIF → (III) formulation dispersed in reservoir → (IV) addition of porcine lipase to dispersion reservoir. The red solid lines show changes in frequency (Hz, left y-axis) over time, and dashed blue lines show changes in dissipation (10^{-6}, right y-axis) over time.

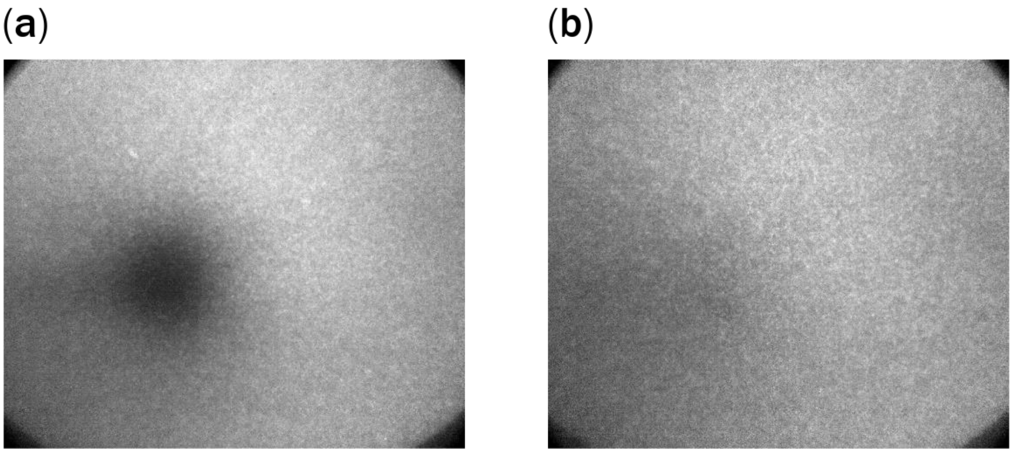

Figure S4. (**a**) TIRF image of photobleached FRAP spot in hydrated LiDo membrane, 1 sec after laser illumination. (**b**) Recovered FRAP spot, 60 sec after laser illumination.

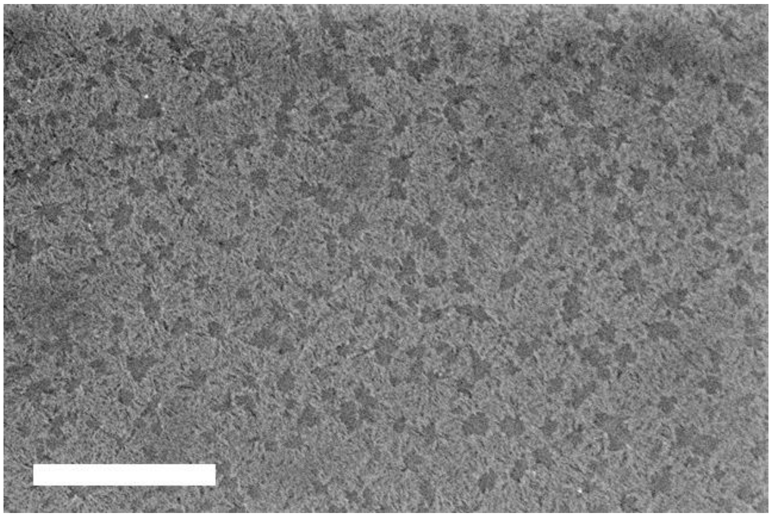

Figure S5. Scanning electron micrograph of the PVDF thin film. Scale bar represents five µm.
